# Therapeutic and Diagnostic Translation of Extracellular Vesicles in Cardiovascular Diseases: Roadmap to the Clinic

**DOI:** 10.1161/CIRCULATIONAHA.120.049254

**Published:** 2021-04-06

**Authors:** Susmita Sahoo, Marta Adamiak, Prabhu Mathiyalagan, Franziska Kenneweg, Sabine Kafert-Kasting, Thomas Thum

**Affiliations:** 1 Cardiovascular Research Institute, Icahn School of Medicine at Mount Sinai, New York (S.S., M.A., P.M.).; 2 Institute of Molecular and Translational Therapeutic Strategies (IMTTS) (F.K., S.K-K., T.T.), Hannover Medical School, Germany.; 3 REBIRTH Center for Translational Regenerative Medicine (T.T.), Hannover Medical School, Germany.; 4 Fraunhofer Institute for Toxicology and Experimental Medicine, Hannover, Germany (S.K-K., T.T.).

**Keywords:** biomarkers, cardiovascular diseases, extracellular vesicles, exosomes, therapeutics

## Abstract

Exosomes are small membrane-bound vesicles of endocytic origin that are actively secreted. The potential of exosomes as effective communicators of biological signaling in myocardial function has previously been investigated, and a recent explosion in exosome research not only underscores their significance in cardiac physiology and pathology, but also draws attention to methodological limitations of studying these extracellular vesicles. In this review, we discuss recent advances and challenges in exosome research with an emphasis on scientific innovations in isolation, identification, and characterization methodologies, and we provide a comprehensive summary of web-based resources available in the field. Importantly, we focus on the biology and function of exosomes, highlighting their fundamental role in cardiovascular pathophysiology to further support potential applications of exosomes as biomarkers and therapeutics for cardiovascular diseases.

The last decade has seen a sharp increase in the number of scientific publications describing the physiological, pathological, and therapeutic functions of extracellular vesicles (EVs). According to the minimal information for studies of extracellular vesicles guidelines set by the International Society for Extracellular Vesicles (ISEV) for EV studies,^1^ EV is a collective term that describes various subtypes of naturally released membranous structures from cells that are delimited by a lipid bilayer and cannot replicate (ie, they do not contain a functional nucleus). EVs are gaining popularity in clinics as therapeutic drug delivery vehicles that transfer bioactive molecules such as proteins, genes, microRNAs (miRNAs), viruses, and other therapeutic agents to treat diseases and halt disease progression. Despite our limited but growing knowledge of EV biology and technology, it is imperative to understand their fundamental role in cardiovascular pathophysiologies to engineer the complex mechanisms of EV cargo sorting in pursuit of designing next generation EV-based therapeutic delivery systems.

We discuss recent advances and challenges in exosome research with an emphasis on scientific innovations in isolation, identification, and characterization methodologies, and we provide a comprehensive summary of web-based resources available in the field. Importantly, we focus on the biology and function of exosomes, highlighting their fundamental role in cardiovascular pathophysiology to further support potential applications of exosomes as biomarkers and therapeutics for cardiovascular diseases.

## The Vesicular World

### Classification of EVs

**Figure 1.**
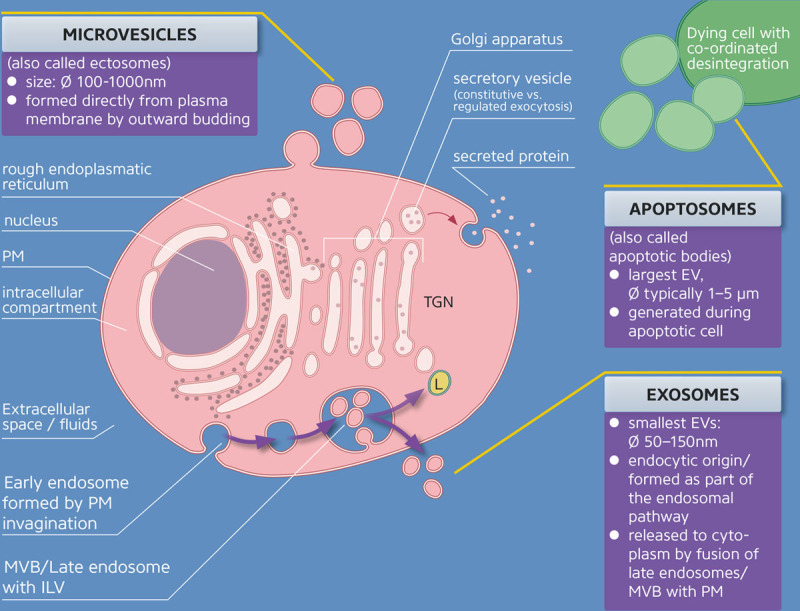
**Formation, characterization, and secretion of various extracellular vesicles such as microvesicles, exosomes, and apoptosomes.** Blue indicates extracellular compartment; red indicates intracellular compartment; and yellow indicates acidic compartment. Note that organelle proportions are not to scale. EV indicates extracellular vesicles; ILV, intraluminal vesicles; L, lysosome; MVB, multivesicular bodies; PM, plasma membrane; RER, rough endoplasmic reticulum; and TGN, trans-Golgi network.

EV populations released from cells as well as found in circulating biofluids are known to be heterogeneous in their origin, size, and composition.^2^ There is no gold standard for EV nomenclature that currently exists. However, a general consensus is that some EVs that are released directly from a cell’s plasma membrane are often called microvesicles or ectosomes. These EVs display sizes ranging from several nanometers to microns. Internal vesicles generated in multivesicular endosomal compartments are termed as exosomes, which are secreted by fusion of the multivesicular body membrane with plasma membrane. Exosomes are classically defined by their size (generally <150 nm in diameter) and contain endosome-associated proteins.^2^ However, the term exosomes is more commonly used in the literature, as well as in this article, for a heterogenous population of small-size EVs isolated by either ultracentrifugation methods or size-exclusion filters without demonstrating their intracellular origin. Exosomes themselves are also considered to be a very heterogenous population. Interestingly, an abundant population of nonmembranous (nonvesicular) nanoparticles known as exomeres (≈35 nm) was recently discovered.^3,4^ Microvesicles are larger (≈100 nm and larger) plasma membrane–derived ectosomes. Apoptoic bodies originate during apoptosis, and are also large vesicles (≈50–500 nm).^5–9^ In this review, we focus on exosomes and microvesicles as major groups of EVs and as potential therapeutic vehicles to treat cardiovascular disease. A sketch of exosome biogenesis and their basic characteristics is provided in Figure 1, in comparison to other extracellular vesicles such as microvesicles^10^ and apoptosomes.^11^

### Exosomes: Crucial Members in Cardiovascular Signaling

Given growing interest in the role of exosomes in cardiovascular physiology and pathology, here we summarize the most recent findings on the role of exosomes as signaling mediators, biomarkers, and potential therapeutic targets that are relevant to cardiovascular medicine. Furthermore, this review provides a comprehensive update on recent methodological advances in the study of exosomes and intends to be valuable to researchers currently active in exosome research, as well as to recent scientific newcomers.

However, before exploring different aspects of exosomes, it is worth explaining why exosomes earned so much interest in the cardiovascular field:

1. Exosomes, a subgroup of EVs, are nanosized membrane particles actively released by cells under both physiological and pathological conditions.
2. Exosomes mediate intercellular transport of cytosolic cargo.
3. Exosomes offer great potential to the clinical field, with applications in both diagnostics and therapeutics.

## New Horizons in Exosome Isolation

### A Beginner’s Guide to Exosome Isolation

Small size and quantity, varying physicochemical properties, and complexity of the surrounding biological fluid make exosomes difficult to obtain in relatively pure preparations. Separation of exosomes from other extracellular entities and vesicle subtypes represents a significant challenge mainly because of the lack of universal or unequivocally specific exosome-based markers.^12^

A plethora of different isolation methods has been described that impact various degrees of EV purity, integrity, and active state, as well as yields (Table 1).^13^ Because there is no single optimal separation method for exosome isolation or characterization fulfilling experimental and clinical needs, choosing a suitable method for exosome studies could be a challenging decision.

**Table 1.**
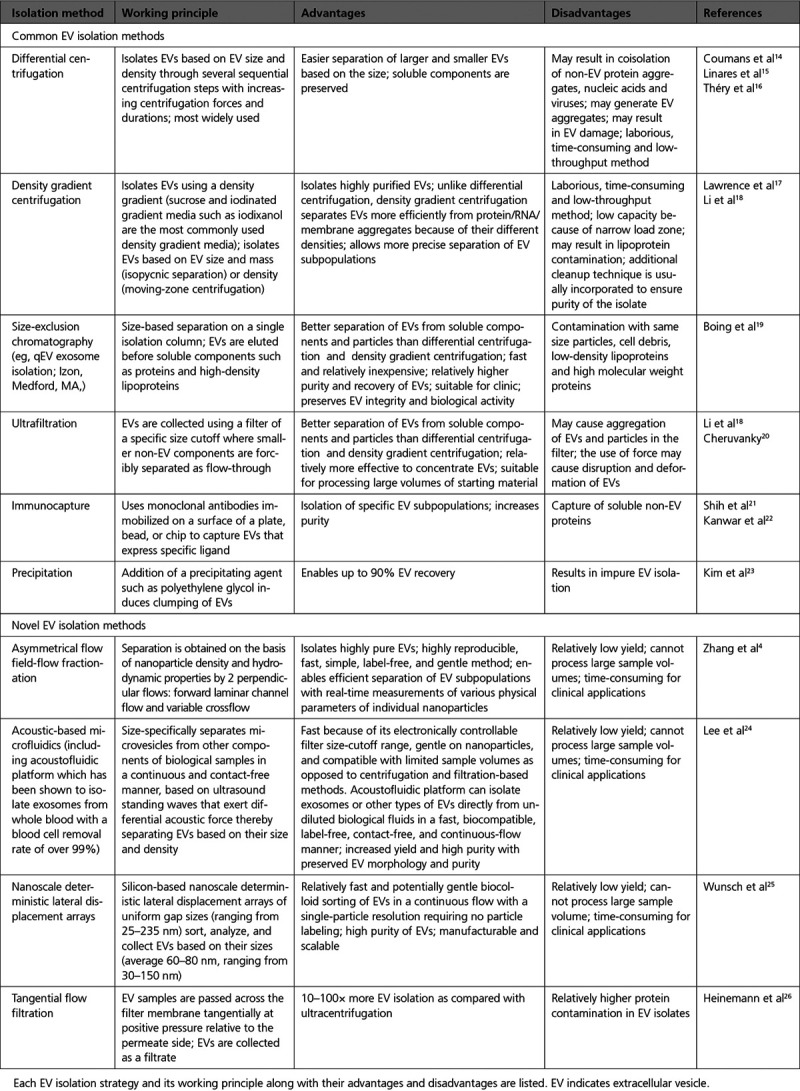
List of EV Isolation Methods

#### Do Not Follow the Beaten Track: Considerations in Selecting an Isolation Method

The choice of separation method should be guided by the type of exosome-containing matrix and required degree of exosome yield, purity, integrity, and concentration that is dictated by the downstream applications and scientific question to be addressed.^27^

##### Know Your Sample and Beware of Contaminants

Regardless of the type of starting material (biological fluid, cell culture conditioned media, or tissue), similar preanalytical elementary considerations apply. For example, it is important to ensure that separated exosomes are indeed from extracellular space rather than from intracellular space or soluble nanoparticles released as a result of exosome-containing matrix harvest, processing, and storage. However, each exosome-containing matrix presents specific biophysical and chemical characteristics, thereby requiring additional sample collection and processing considerations. For instance, it is especially important for cellular studies to avoid contamination of cell-derived vesicles by serum exosomes, microbes (eg, mycoplasma), or dead cell–derived vesicles.^1,28^ By contrast, for exosome isolation from biofluids, in most cases, dilution of biological fluids may be advisable and may enhance the recovery of vesicles.^28^ Additionally, one should be aware that vesicles recovered from biofluids will not only have a mixed cellular origin, but also will likely be contaminated to various degrees with nonexosomal structures (eg, fat-containing vesicles) and more advanced isolation methods should be used to separate these components from exosomes.^1^

##### Think Downstream

Finally, the choice of isolation procedures is additionally influenced by the ultimate goal of exosome research question. The type of study—discovery, clinical, or preparative research—will mandate a different level of yield, purity, and concentration depending on the application, thereby requiring specific isolation approach. In some cases, combination of several methods or specific modifications to existing methods may offer better outcomes when addressing specific types of scientific questions.

### The Gold Standard and Beyond

A variety of exosome isolation methods are currently in use, each with its own advantages and disadvantages. While centrifugation-based and size-based exosome isolation methods are traditionally followed by wide range of researchers, rapid advances in modern science and technology have started to yield many new techniques for improving exosome isolation, both quantitatively and qualitatively.

In Table 1, we discuss some of the most commonly used EV isolation methods, along with the advantages and disadvantages of each technique. We also introduce several innovative exosome isolation methods (eg, asymmetrical flow field-flow fractionation, acoustic-based microfluidics) that are currently being developed, some of which may become more prominent in the coming years if they achieve better recovery and purity.

#### The Unimaginable Becomes Reality: Isolation of Exosomes From Tissues

To date, isolating exosomes directly from tissues remains a major challenge mainly attributed to the lack of a suitable exosome isolation procedure as well as the complexities associated with isolating exosomes with preserved biochemical properties from the intact extracellular milieu.

##### Homogenization

By using tissue homogenization, exosomes have been isolated from tissues of the mouse brain, freshly removed^29^ or frozen postmortem,^29^ and central nervous system.^30^ However, it should be noted that tissue homogenization may lead to physical damage and compromise the extracellular environment with intracellular vesicles and exosome mimetics.^29,30^

##### Enzyme-Based Mild Tissue Dissociation

In 2017, Vella et al reported an efficient protocol for the enrichment of exosomes from postmortem brain tissues stored at −80°C which provided significant advantages over previously described protocols.^31^ In this study, the purification of exosomes was achieved by gentle dissociation of the tissue using collagenase type III to free the brain extracellular space, followed by sequential low-speed centrifugations and sucrose cushion ultracentrifugation.^31^

##### Tissue Disruption

In relation to the cardiac research field, Loyer et al recently demonstrated that small and large EVs can be isolated directly from sham-operated and infarcted mouse heart tissue as well as human heart tissue of patients undergoing aortic valve replacement by tissue disruption and different centrifugation steps.^32^

#### Clinical-Grade Exosomes

A notable challenge in the advancement of exosome-based therapeutics into clinical applications lies in developing sterile, scalable, reproducible, and efficient protocols for clinical-grade exosome production with minimal batch-to-batch variation.^33,34^

The process and feasibility of generating good manufacturing practice−grade exosomes for therapies was recently demonstrated using bioreactor-based culture system.^35^ In that study, isolation and purification of exosomes were achieved by filtration and ultracentrifugation using closed and semiclosed systems followed by exosomes analyzed by NanoSight and flow cytometry and tested for endotoxin and sterility to ensure their potential clinical applicability.^35^ Similar developments are currently emerging to successfully upscale good manufacturing practice−grade manufacturing methods for the treatment of cardiovascular and other diseases.^36^

## Deep Dive Into Exosome Biology

### Exosome Composition: Piece by Piece

Genuine and unique markers that distinguish different EV subpopulations and allow for precise analyses of their subtypes are still not well-established. Nevertheless, a common feature of all EVs is a lipid bilayer membrane that surrounds a rich cargo of selective biomolecules from its originating cells. Thus, the EV cargos are partially reflective of their cellular origin.

#### Proteins

An important criterion for EV subtype classification is to determine EV protein markers, which can be membrane-bound or present in the EV transmembrane or as intraluminal soluble proteins.

##### Unique or Enriched?

Biochemical studies by Kowal et al revealed some of the common proteins shared by several EV subtypes that include: actin, ezrin, moesin, HSC70 (heat shock cognate 71 kDa), HSP70 (heat shock protein 70), flotillin-1, MHCI (major histocompatibility complex class I), MHCII (major histocompatibility complex class II), annexin II, CD63 (cluster of differentiation 63), and CD9 (cluster of differentiation 9).^2^ Interestingly, small EVs are selectively enriched with unique proteins such as TSG101 (tumor susceptibility 101), syntenin-1, EHD4 (Eps15 homology domain protein 4), annexin XI, and ADAM10 (disintegrin and metalloproteinase domain-containing protein 10), and could be distinguishable from larger EVs.^2^ Conversely, actinin-4, mitofilin, and GP96 (heat shock protein 96) were shown to be unique or enriched in large EVs.^2^ Additionally, Jeppesen et al recently performed extensive proteomic profiling of small EVs and nonvesicular fractions, and identified annexin A1 as a specific marker for microvesicles that are shed directly from the plasma membrane.^3^

##### Hunt for Exosomal Markers

Although numerous proteomic studies have implicated Alix (programmed cell death 6-interacting protein), TSG101, CD63, and CD9 as exosomal markers^37^ because of their increased enrichment in exosomes; these markers are not to be considered as canonical exosome markers.^12^ Indeed, using global integrated proteogenomic analysis of neuroblastoma cell–derived EVs, Keerthikumar et al identified enrichment of Alix, TSG101, CD63, and CD9 in exosomes subpopulations as compared with ectosomes.^38^ Similarly, another study supported that CD81 (cluster of differentiation 81) might specifically be utilized as an exosomal marker.^39^ Together, these studies indicate that selectively enriched proteins could represent specific EV subtypes and serve as biomarkers for both investigations as well as for diagnostics.

#### RNA

While the occurrence of proteins in EVs was reported together with the discovery of EVs,^40^ the presence of RNA in EVs was only demonstrated in the last 2 decades.

##### It’s All About the Copy Number

In 2007, Valadi et al were the first to report the existence of mRNAs and miRNAs in EVs derived from mast cells and the functional transfer of RNA to EV-targeted cells.^41^ Several years later, Nolte-’t Hoen et al found a large variety of small noncoding RNA (ncRNA) species in immune cell–derived vesicles, including small nucleolar RNA, Y RNA, mitochondrial RNA, and vault RNA as well as mRNA (mostly fragmented).^42^ According to Wei et al, exosomes are enriched in small ncRNAs, such as miRNAs and precisely processed tRNA and Y RNA fragments.^43^ Even though exosomal RNA composition is an expanding and promising area of research, recent studies suggest that massive exosome uptake would be required to ensure functional impact of transferred RNA on recipient cells as there seems to be <1 copy of nonribosomal RNA per exosome.^43^ However, studies of the cardiovascular system showed that also small miRNAs can be shuttled between cardiac cell types (endothelial cells, cardiomyocytes, fibroblasts) via exosomes.^44,45^

##### Focus on Long ncRNAs

Interestingly, long ncRNAs (lncRNAs) are often detected in EVs and thought to act as messengers in intercellular communication.^46^ One of the first observations of this phenomenon was made by Kogure et al where a 1198-nucleotide ultraconserved lncRNA referred to as TUC339 was identified in exosomes derived from hepatocellular carcinoma and its enrichment was dramatically altered within EVs compared with that of cells of their origin.^46^ Additionally, in these studies Kogure et al demonstrated that hepatocellular carcinoma cell–derived EVs can be taken up by other hepatocellular carcinoma cells resulting in intercellular transfer of ultraconserved lncRNA with subsequent modulation of cell growth and spread.^46^ In hypoxic cardiomyocytes, RNA sequencing of lncRNAs revealed strong differences in cargo of exosomes in contrast to larger microvesicles or even complete cells.^47^ Recently, Li et al showed for the first time the presence of another type of endogenous lncRNA species, circular RNA (circRNA), in exosomes derived from MHCC-LM3 liver cancer cells.^48^ RNA sequencing (RNA-seq) analyses revealed that circRNAs were highly enriched in exosomes compared with parental cells. Interestingly, the authors found that serum exosomal circRNAs may distinguish patients with cancer from healthy individuals demonstrating its substantial translational potential as a circulating biomarker for cancer diagnosis.^48^

##### Modified RNA in EVs?

Recent studies provide evidence that EV RNAs are not just linear RNA molecules, but rather they can be modified. Specifically there is evidence for EV-specific tRNA modification, perhaps indicating a role for posttranscriptional modification in the sorting of some RNA species into EVs. Moreover, the RNA-binding protein YBX1 (Y box binding protein 1), which is required for the sorting of selected miRNAs into exosomes, plays a role in the sorting of highly abundant small ncRNA species, including tRNAs, Y RNAs, and vault RNAs.^49^ These observations open up an interesting line of investigation into the extent and purpose of RNA modifications in EVs and their roles in cardiovascular physiology and pathology.^50^

#### Lipids

To a large extent, EVs constitute of membrane lipids organized in a bilayer structure. In addition to the lipid carriers such as fatty acid binding proteins embedded in the bilayer, EVs also shuttle various bioactive lipids, which are captured from the cytosol during exosome formation.^51–53^ EVs are often enriched in eicosanoids, fatty acids, and cholesterol, and their lipid composition can be modified by in vitro manipulation.^51–53^ EVs also contain metabolizing enzymes involved in lipid synthesis so they can represent an autonomous production unit of bioactive lipids.

##### Don’t Underestimate EV Lipids in Pathologies

Compared to their parental cells, EVs are 2 to 3 times enriched in cholesterol, sphingomyelin, phosphatidylserine, and glycosphingolipid, and the accumulation of these EV-components in cells might modulate recipient cell homeostasis.^54–56^ For instance, Hough et al demonstrated that chronic airway inflammation may be driven by alterations in the composition of lipid mediators within airway EVs of human subjects with asthma.^57^ Data of diagnostic relevance was recently shown by Pieragostino et al, who reported a high number of acid sphingomyelinase–enriched exosomes in multiple sclerosis, opening a window for novel therapeutic approaches in the treatment of this disease that attacks the central nervous system.^58^

##### What If It Can Be Quantified?

As lipid bilayers are indispensable components of EVs, technological developments in EV lipidomics may further contribute to developing a reliable and reproducible method for the standardization and characterization of EV samples and/or EV subtypes. Osteikoetxea et al and Visnovitz et al proposed the use of total lipid content and the protein-to-lipid ratio as additional parameters to total protein content and particle counts for quantifying and characterizing EV preparations. Authors have also proposed that protein-to-lipid ratio, lipid bilayer order (which reflects the degree of lipid packing), and selected lipid composition can also discriminate among EV subpopulations of a different size.^59,60^

##### The Emerging Importance

The lipid composition of EVs attracts substantial attention not only because of the proposed role of these molecules in various biological processes but also because of their potential to serve as biomarkers for a variety of diseases, including cancer. Skotland et al recently investigated the lipid content of exosomes purified from the urine of 15 patients with prostate cancer and 13 healthy volunteers, and identified molecular lipid species in urinary exosomes as potential prostate cancer biomarkers.^51^

#### The Known, The New, and a Possible Surprise: Unique Exosome Cargo

##### Viruses and Viral Components

Exosomes are also produced and secreted by virus-infected cells and are believed to be involved in cell-to-cell communication between infected and uninfected cells.^61^ Adeno-associated virus (AAV) is one of the most actively investigated gene therapy vehicles because of the vector’s good safety profile in clinical trials. However, one significant obstacle to AAV-based gene therapy is the potential blocking of AAV by naturally existing anti-AAV neutralizing antibodies. Recently, it was shown that AAV capsids may be incorporated into EVs during virus vector production and therefore, exosome-associated AAV (Exo-AAV) can be isolated from conditioned medium of the packaging cell line.^62^ Exo-AAV vectors outperformed conventional AAV vectors in transduction, both in vitro and in vivo, and were more resistant to neutralizing antibodies compared with standard AAV.^63,64^ For example, Hudry et al demonstrated that Exo-AAV is capable of efficient transduction of cells in the central nervous system after systemic injection in mice.^65^ Importantly, first attempts to test the use of Exo-AAV as superior agents for delivering genes (eg, SERCA2a [sarcoplasmic/endoplasmic reticulum Ca^2+^ ATPase 2a]) to cardiomyocytes and to the heart were made in our laboratory and showed that Exo-AAV9 outperformed conventional AAV vectors in preserving cardiac function post–myocardial infarction, even in the presence of neutralizing antibodies^66^ (and unpublished data).

##### Genomic and Mitochondrial Components

###### Mitochondrial DNA.

EVs have been previously shown to deliver genomic DNA to target cells.^67,68^ Additionally, several studies demonstrated that cells release vesicles containing mitochondrial DNA.^69–72^ For example, Sansone et al identified the full mitochondrial genome packaged in cancer-associated fibroblast-derived EVs and in circulating EVs from patients with hormonal therapy–resistant metastatic breast cancer.^72^ Additionally, authors demonstrated that transfer of mitochondrial DNA through EVs acts as an oncogenic signal promoting an exit from dormancy of therapy-induced cancer stem-like cells and leading to endocrine therapy resistance in breast cancer.^72^

###### Mitochondrial Proteins.

Recent studies also suggest that in addition to mitochondrial DNA, mitochondrial proteins are also secreted via EVs.^71,73^ Using EV membrane isolation and mass spectrometry analysis, Jang et al discovered enrichment of mitochondrial membrane proteins in the melanoma tissue–derived EVs, compared with nonmelanoma-derived EVs. Specifically, EVs with mitochondrial inner membrane proteins MT-CO2 (mitochondrially encoded cytochrome c oxidase I) and COX6c (cytochrome c oxidase subunit 6C) were detected in the plasma of melanoma patients as well as in ovarian and breast cancer patients. Importantly, this subpopulation of EVs was shown to contain active mitochondrial enzymes.^73^

###### Always Double-Check.

Although exosomes are thought to be mediators of extracellular DNA secretion, Jeppesen et al recently provided evidence that double-stranded DNA and DNA-binding histones are not carried by exosomes or any other type of small EVs.^3^ Therefore, some precautions, such as determining whether or not the detected DNA or protein is truly carried by EVs rather than being an artefactual contaminant, are advisable for such studies.

### It’s All About Reaching the Target: Exosome Uptake

#### The More, the Better: Mechanisms of Exosome Uptake

Exosomes transfer their molecular cargo to recipient cells to regulate cell function. Several types of interactions may be involved in the functional communication of exosomes with cells, including: release of exosome contents in the extracellular space; exosome-binding to the recipient cell surface; exosome–plasma membrane fusion; direct transfer of cargo through connexin 43–containing channels formed by the docking of exosomes and target cells; and uptake by endocytosis.^7,74^ Exosomes have been shown to be internalized by recipient cells by a variety of endocytic pathways, such as clathrin-dependent endocytosis and clathrin-independent pathways (eg, caveolin-mediated uptake), macropinocytosis, phagocytosis, and lipid raft–mediated internalization.^75^ Interestingly, it seems possible that a heterogeneous population of vesicles may gain entry into a cell via >1 route. For functional implications, it is important that the vesicle is uptaken and not simply fated for the lysosome thus destroying the signal. Importantly, the vesicle has to be uptaken unpackaged and the signal should be delivered more specifically.

#### Friend or Foe: Cell Type–Specific Exosome Uptake

Contradictory opinions as to the mode of exosome internalization exist within the literature. Results from some studies show that exosomes can be taken up by essentially every cell type investigated,^76^ while other studies demonstrate that vesicular uptake is a highly specific process between a specific cell type and EVs.^77^

Zech et al showed that pancreatic adenocarcinoma–derived exosomes bind to, and are taken up by, all leukocyte subpopulations both in vivo and in vitro. However, these leukocyte subpopulations differed in tumor–exosome uptake, which was highest for peritoneal exudate cells and lowest for granulocytes.^76^ In another study, tetraspanin 8–containing lymph node stroma–derived EVs were most effectively internalized by endothelial cells and pancreatic cells but to a lower degree by parental lymph node stroma cells.^78^ Näslund et al demonstrated that exosomes from breast milk can be taken up via monocyte-derived dendritic cells partly via MUC1 (mucin 1, cell surface–associated)/DC-SIGN (dendritic cell–specific intercellular adhesion molecule-3–grabbing nonintegrin) interaction, whereas plasma exosomes lacking MUC1 are unable to enter these cells.^79^ Moreover, results from our laboratory indicate that injection of mouse hindlimb ischemic tissue with exosomes derived from human CD34^+^ stem cells resulted in the most efficient exosome uptake by endothelial cells as compared with smooth muscle cells and fibroblasts.^77^

#### Seeing Is Believing: Methods for Visualization of Exosome Uptake

A major challenge for the development and optimization of exosome-based diagnosis and treatment is being able to visualize clinical and preclinical in vivo transfer of exosomes to recipient cells. To address this problem, a few groups have recently developed novel approaches based on genetic modification of the cell of their origin, which then secrete EVs containing labeled components.^80^ A labeling approach is very important, especially from a clinical drug development perspective, to gain more information about pharmacokinetics and biodistribution of EVs. Here, we discuss examples of such strategies.

##### Fluorescent or Bioluminescent Exosomes

Genetic fusion of fluorescent protein (eg, red fluorescent protein [tdTomato]) to a consensus palmitoylation sequence induced its localization at the plasma membrane, as well as in secreted EV subpopulations. Bound or internalized EVs were then visualized by intravital microscopy of tumor tissue in animals with tumors expressing this fusion protein.^81^ Fusion of luciferase to a protein transmembrane domain similarly allowed its secretion in EVs and subsequent analysis of distant bioluminescent cells in vivo.^82^ Authors also designed an intracellular probe to fluorescently label mRNA secreted in EVs and tools to detect EV-associated transcripts encoding luciferase signal in target cells.^81,82^

##### Green Fluorescent Protein–Tagged Human CD63 Transgenic Animal Model

Another elegant approach to elucidate the intercellular EV transfer in vivo was proposed by Yoshimura et al,^83^ where the authors generated a novel transgenic rat model in which green fluorescent protein (GFP)–tagged human CD63 (CD63-GFP) is under control of the CAG (cytomegalovirus early enhancer/chicken β actin) promoter, resulting in high expression of GFP in various body tissues. Indeed, exogenous human CD63-GFP was detected on EVs isolated from 3 body fluids of animals: blood serum, breast milk, and amniotic fluid. Additionally, in vitro analyses demonstrated transfer of serum-derived CD63-GFP–expressing EVs into recipient rat embryonic fibroblasts, where the EVs localized in endocytic organelles. These findings suggest that transgenic animal model developed by Yoshimura et al may provide significant information for understanding of the intercellular transfer as well as mother-to-child transfer of EVs in vivo.

##### CD63-pHluorin

In a recent article, Verweij et al provided a unique model to track in vivo interorgan communication by endogenous exosomes at the individual vesicle level with high spatiotemporal accuracy.^84^ The authors have successfully live-visualized single endogenous vesicles in the whole zebrafish embryo using a fluorescent reporter, CD63-pHluorin. This model will allow investigation of the composition of EVs and the molecular mechanisms controlling their biogenesis, fate and function in receiving cells.

### Beauty and Strength in Heterogeneity

#### The Apple Falls Far From the Tree: Distinct Content Between Exosomes and Cells of Origin

Interestingly, EV contents are not always reflective of the cell of their origin.^85–87^ Recently, it has been shown that certain miRNAs are differentially expressed in mouse induced pluripotent stem cells (iPSCs) and iPSC-derived EVs.^85^ Indeed, using quantitative polymerase chain reaction–based method 33 miRNAs were detected only in iPSC-derived EVs, which suggests their selective enrichment during EV production and release by iPSCs.^85^ Similar phenomenon was previously observed by Shurtleff et al, who performed Illumina-based small RNA-seq on libraries prepared from purified exosomes and their parent cell of origin.^87^ They found at least 134 miRNAs that are selectively enriched in exosomes as compared with parent cells.^87^ Similarly, Squadrito et al showed that several miRNAs are differentially enriched in macrophage-derived exosomes as compared with their parent cells.^86^ The same strong differences in the cargo between different extracellular vesicles was shown for lncRNAs.^47^

Nonetheless, the underlying mechanisms of the active sorting of specific miRNAs to EVs and the significance of miRNA transfer to recipient cells are still poorly understood. Some studies propose that RISC (RNA-induced silencing complex)–associated proteins are involved in the loading process of miRNAs into EVs as RNA-induced silencing complex proteins were found to be colocalizing with multivesicular bodies where exosomes are sorted for packaging and release.^88^ Other studies reported that the neutral sphingomyelinase 2 might in part regulate the sorting process of specific miRNAs into small EVs.^89^ In addition, posttranscriptional modifications such as 3´-end uridylation were previously shown to play a role in defining the fate of miRNAs to be loaded into EVs.^90^ Villarroya-Beltri et al described another mechanism where the researchers discovered that RNA-binding proteins (RBPs) recognize specific GGAG motifs in the 3´-end of miRNAs and regulate miRNA packaging into exosomes.^91^ Specifically, they identified that binding of hnRNPA2B1 to exosomal miRNAs is dependent on its sumoylation state.^91^ Another example of RNA-binding protein–dependent miRNA sorting is the identification of Y-box protein 1 as a candidate exosomal miR-223 sorting protein.^87^

These findings open the way for a great tool to control the specific miRNA content in EVs by either mutagenesis of sequence motifs or modulation of hnRNPA2B1 sumoylation and expression of RNA-binding proteins. However, it remains unclear whether these proteins only control a specific set of miRNAs or are globally involved in the RNA sorting process, nor is it confirmed if RNA-binding proteins play an actual active role or are just passively attached to EV sorting process. Indeed, direct and persuasive evidence for a physiological role of EV-borne miRNAs has so far proven elusive. Alternatively, it seems possible that vesicles may be a convenient carrier to eliminate unnecessary or inhibitory RNA molecules from cells.^86^

#### Distinct Molecular and Biological Properties of EV Subpopulations

Recently, several studies have investigated the molecular composition of different EV subpopulations from a single cell type. Interestingly, these direct comparisons evaluated that vesicular subtypes have at least partly different protein, lipid, and RNA content, and might exert different biological functions on target cells.^2,92–95^

The results obtained by Collino et al demonstrated heterogeneity in quantity and composition (ie, exosomal markers and miRNA content) of subpopulations distributed at different densities.^93^ Authors demonstrated that the population of EVs contained in the medium floatation density fraction showed superior activity in promoting renal protection from injury in vitro.^93^ Tauro et al used magnetic beads coated with anti-A33 and anti-EpCAM (epithelial cell adhesion molecule) antibodies to isolate 2 molecularly distinct populations of exosomes, EpCAM- and A33-Exos, being released from the apical and basolateral side of human colon carcinoma cells, respectively.^96^ These findings suggest that vesicles with different molecular structure can be released from different cell regions (eg, polarized intestinal epithelial cell surfaces) and that the expression of plasma membrane molecules can be used to separate EVs, which would be coisolated using other purification methods.

## Tools for Accelerating Exosome Research

The purpose of this section is to list and make readers aware of a variety of available web-based resources that integrate our knowledge of exosome biology and provide databases of different vesicular molecules. Best practices for biological sample collection and analysis of submicron particles, including exosomes and other EVs, have been discussed in several position papers and statements from ISEV which we also summarize in this section.

### Web-Based Resources

#### Vesiclepedia

Vesiclepedia (http://microvesicles.org) serves as an online, community-annotated compendium of molecular data (mRNAs, miRNAs, proteins, and lipids) pertaining to EVs from >500 independently published studies.^97^ It catalogs recent studies on all classes of EVs, including apoptotic bodies, exosomes, large dense-core vesicles, microparticles, and microvesicles. EV researchers can query molecules of interest or browse by organism, vesicle type, molecular content type, or sample material.

#### ExoCarta

ExoCarta (http://www.exocarta.org) is a database manually curated by EV biologists, which serves as a free web-based compendium of molecules identified in exosomes derived from multiple cell types.^98–101^ The current version of ExoCarta hosts ≈41 860 protein, 4946 mRNA, and 1116 lipid entries catalogued from 286 exosomal studies from 10 different species (including 6514 molecules identified in *Homo sapiens* and 1714 in *Mus musculus*), and annotated with ISEV minimal experimental requirements for definition of EVs.^101^ ExoCarta can be queried using the gene symbol, protein name, and miRNA ID, or it can be browsed as a group based on the organism, molecular content type, sample material, and gene symbol. Furthermore, ExoCarta features dynamic protein–protein interaction networks and biological pathways of exosomal proteins.^101^

#### EVpedia

EVpedia (http://evpedia.info) is an integrated and comprehensive proteome, transcriptome, and lipidome database of prokaryotic and eukaryotic EVs.^39,102,103^ EVpedia provides a broad array of tools, such as (1) search and browse tools for vesicular proteins, mRNAs, miRNAs, lipids, and metabolites, as well as publications and principal investigators; (2) a database for identification of orthologous vesicular components; (3) tools for bioinformatic analyses including Gene Ontology enrichment analysis and network analysis; and (4) personalized function to deposit the user’s own datasets, results, and literature of interest.^39^ Recently, EVpedia has been further improved (eg, by addition of *Top 100+ EV* markers list). Now EVpedia includes 14 192 publications, 722 551 vesicular components from 503 high-throughput studies (specifically 524 027 proteins; 94 355 mRNAs; 64 785 miRNAs; 3744 lipids; and 848 metabolites from *Eukaryotes*) and has been globally accessed >19 500 000 times from >88 countries. In addition, about 2445 members from 78 countries have participated in developing EVpedia.

#### exoRBase

exoRBase (http://www.exoRBase.org) is a web-accessible database that is a repository of circRNA, lncRNA, and mRNA derived from RNA-seq data analyses of exosomes derived from human blood. The first release of exoRBase featured 58 330 circRNAs, 15 501 lncRNAs, and 18 333 mRNAs.^104^ Furthermore, 77 experimental validations from 18 published articles are also included.^104^ exoRBase contains the integration and visualization of RNA expression profiles based on normalized RNA-seq data from healthy individuals as well as from patients with different diseases.

#### ISEV Massive Open Online Course

The ISEV Education Committee has produced a massive open online course for researchers interested in the field of EVs. The first ISEV massive open online course, *Basics of Extracellular Vesicles*, can be accessed via the Coursera platform (https://www.coursera.org/learn/extracellular-vesicles) and is the result of collaboration with University of California Irvine (USA), University of Gothenburg (Sweden), and Pohang University of Science and Technology (South Korea). The goal of the course is to provide the basic knowledge about EV history, nomenclature, biogenesis, and EV cargo, as well as the release and uptake mechanisms, collection, and processing before isolation, different isolation methods, characterization. and quantification techniques.

#### EV-TRACK Platform

EV-TRACK (http://evtrack.org) is an open-source knowledgebase that integrates methodological parameters of EV-related publications with the goal of motivating researchers, reviewers, editors, and funders to put experimental guidelines into practice and facilitating standardization of EV research through systematic reporting on EV biology and methodology.^105^

#### exRNA Atlas

exRNA Atlas (https://exrna-atlas.org) is the data repository of the Extracellular RNA Communication Consortium^106^ and provides access to small RNA–seq and quantitative polymerase chain reaction–derived extracellular RNA (exRNA) profiles from multiple human and mouse biofluids (cerebrospinal fluid, serum, plasma, saliva, and urine). A suite of web-accessible analysis and visualization tools enables users to analyze exRNA-seq profiles from the Atlas, process and analyze their own exRNA-seq data, and contribute their data and analyses to the Atlas. In an attempt to minimize experimental variability, all exRNA-seq datasets are uniformly processed using the exceRpt (extra-cellular RNA processing toolkit) pipeline^107^ and Extracellular RNA Communication Consortium–developed quality metrics are uniformly applied to these datasets. The latest release of the Atlas (v. 4P1; https://exrna-atlas.org/exatv4p1) contains 2270 exRNA-seq and 3039 quantitative polymerase chain reaction profiles from 19 different studies that cover 23 health conditions, and additionally combines the exceRpt pipeline with computational deconvolution step to facilitate interpretation of exRNA profiling studies and our understanding of exRNA communication.^108^

### ISEV Position Papers and Statements

#### Minimal Information for Studies of Extracellular Vesicles Guidelines

To improve the reliability and credibility of results obtained by individual laboratories, minimal information for studies of EVs guidelines for the field were published,^12^ providing researchers with a set of biochemical, biophysical, and functional standards that should be used to attribute any specific molecular content or functions to EVs. The minimal information for studies of extracellular vesicles 2014 guidelines were updated in 2017^27^ and again in 2018^1^ based on the evolution of our collective knowledge about EVs through the years.

#### ISEV Position Papers

The overview of the current state of knowledge in the field of EV RNA is provided in position statements published in 2013 and 2017.^109, 110^ Both papers discuss EV RNA analysis and bioinformatics, providing detailed guidelines for researchers in the EV field to consider when performing such experiments.^109,110^ Furthermore, published authors are encouraged to make the scientific community aware of possibilities and limitations of approaches currently used in EV RNA research and to call for caution in interpretation of the obtained results.^109^

In the position statement by Lener et al, basic and clinical researchers summarize recent developments and the current knowledge on EV-based therapeutics in clinical trials.^111^ This article highlights various topic-specific aspects (eg, safety and regulatory requirements, production, and quality control processes) that must be considered to develop best practice models for EV therapies and translating EVs into the clinic.

### EV Flow Cytometry Working Group

The EV Flow Cytometry Working Group (http://www.evflowcytometry.org) is a collaborative initiative involving specialists in the fields of EVs, cytometry, and vascular biology to develop more robust flow cytometric methods for the study of submicron particles, including exosomes and other EVs.^112^ To accomplish that, 3 major societies, the International Society for Advancement of Cytometry (ISAC), the International Society on Thrombosis and Haemostasis (ISTH), and ISEV joined forces in 2015 and committed to improve tools, technologies, and standards for EV analysis.

Together, these online sources, as well as the collaboration between scientific communities working on EV biology, provide new opportunities to better communicate and develop EV-based novel therapeutic options.

## Cardiac exosomes: the messengers of health and disease

**Figure 2.**
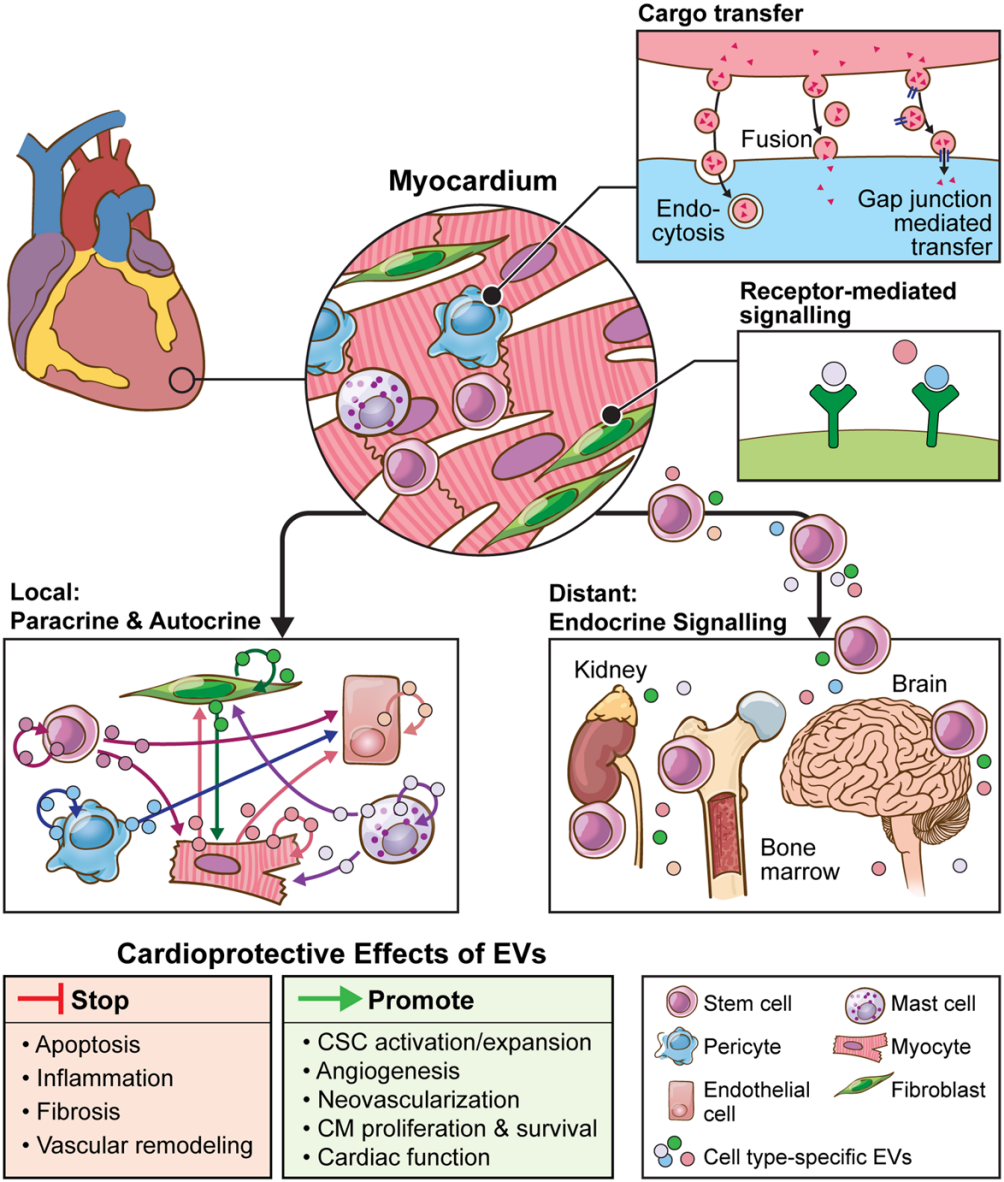
**Mechanisms of EV-mediated local and distal communications of the heart.** Local communication within the heart is mediated by crosstalk between different cell types via EV secretion as indicated by arrows (paracrine manner). Different cardiac cell types secrete EVs that may also function in an autocrine manner affecting the same cell that released the vesicles. On the other hand, distant communication is mediated by secretion of EVs from the myocardium into the systemic circulation, mediating functional crosstalk between the heart and other organs (eg, bone marrow). Myocardial local and distal communications involve exchange of proteins, lipids, and nucleic acids shuttled via EVs between cells through endocytosis, membrane fusion, or gap junction–mediated transfer. Mechanisms by which EVs function additionally include receptor-mediated signaling leading to regulation of transcription and posttranscriptional processes in the target cell. Because of these capabilities, EVs were shown to be effective communicators of biological signaling implicated in multiple processes governing cardiac function under normal cardiac physiology as well as in disease. CM indicates cardiomyocyte; CSC, cardiac stem cell; and EV, extracellular vesicles,

Exosomes were shown to be effective communicators of biological signaling implicated in myocardial function (Figure 2). Here, we describe effects of exosome-derived molecules (mainly microRNAs and proteins) on heart function in health and disease, to support potential applications of cardiac exosomes as biomarkers or therapeutics.

### Cardiac Exosomes at Their Own Playground: Intracardiac Communication

#### Cardiosomes

Exosomes secreted by cardiomyocytes (cardiosomes) exert diverse autocrine and paracrine effects dependent on the conditions of release and therefore, exosomal contents, as well as the type, of a target cell. In their study, Yu et al demonstrated that HIF-1α (hypoxia-inducible factor 1α) is involved in the regulation of hypoxia-induced autocrine effects of TNF-α (tumor necrosis factor α) in cardiomyocytes, mediated by the exosomal pathway.^113^ Recently, Loyer et al showed that small and large vesicles are locally released from the heart in the mouse model of myocardial infarction and that these EVs mainly originate from cardiomyocytes.^32^ Authors further showed that the secretion of EVs regulated the inflammation process postmyocardial infarction through the rapid uptake of EVs by monocytes and subsequent production of IL-6 (interleukin 6) and chemokines CCL-2 and CCL-6 (C-C motif chemokine ligand 2 and 6, respectively).^32^ Interestingly, Ribeiro-Rodrigues et al demonstrated in vitro and in vivo that cardiosomes produced under ischemic conditions stimulate cardiac angiogenesis.^114^ Moreover, they provided evidence that these ischemic cardiosomes are specifically enriched in miR-222 and miR-143 and account for the angiogenic process. More recently, another study identified miR-217 as a promising therapeutic target for congestive heart failure mediating cardiac hypertrophy and cardiac fibrosis processes through regulation of PTEN. Importantly, miR-217 was contained in cardiosomes which were shown to enhance proliferation of fibroblasts in vitro.^115^

#### Fibroblast-Derived Exosomes

Cardiac fibroblast–derived exosomes have been shown to regulate diverse aspects of cardiomyocyte biology such as hypertrophy. For example, Bang et al demonstrated that passenger strand miR, miR-21-3p/miR-21 is significantly enriched in cardiac fibroblast–derived exosomes and regulates hypertrophic crosstalk between cardiomyocytes and cardiac fibroblasts.^45^ Another study has shown that stimulation of cardiac fibroblast with angiotensin II resulted in secretion of exosomes that further induced angiotensin II production and angiotensin II receptor expression in cardiomyocytes and promoted myocyte hypertrophy.^116^

#### Endothelial Cell–Derived Exosomes

There is an extensive literature demonstrating that cardiac endothelial cells are an important source of exosomes within healthy and diseased heart. Endothelial cell–derived exosomes containing miR-214 were shown to suppress senescence and stimulate angiogenic program in target mouse and human endothelial cells.^117^ Interestingly, the 16-kDa N-terminal prolactin fragment, which is associated with peripartum cardiomyopathy, stimulated secretion of exosomal miR-146a from endothelial cells.^118^ Furthermore, these endothelial cell–derived EVs were shown to be taken up by cardiomyocytes, in which exosomal miR-146a mediated posttranscriptional gene silencing of target genes *Erbb4*, *Notch1*, and *Irak1*, and reduced cardiomyocyte metabolic activity.^118^ In another study, EVs secreted by Krüppel-like factor 2–transduced or shear-stress–stimulated human umbilical vein endothelial cells were shown to be enriched in miR-143/145 and to induce atheroprotective phenotype in cocultured smooth muscle cells.^44^ Furthermore, Liu et al provided evidence that clinical and experimental atherosclerotic conditions can promote the packaging of functional miR-92a-3p into endothelial microvesicles, thus promoting angiogenic responses in the recipient endothelial cells by a THBS1 (thrombospondin 1)–dependent mechanism.^119^ Recently, Davidson et al demonstrated that endothelial cell–derived exosomes can confer resistance to simulated ischemia and reperfusion injury in cardiomyocytes via the activation of the ERK1/2 (extracellular signal-regulated protein kinase 1 and 2) MAPK (mitogen-activated protein kinase) signaling pathway, and may contribute to ischemic preconditioning.^120^ In another study, it was shown that endothelial exosomal Mst1 (macrophage stimulating 1) inhibits autophagy and promotes apoptosis in cardiomyocytes under diabetic conditions, and inhibits glucose metabolism by disrupting the binding between Daxx (death domain associated protein) and GLUT4 (glucose transporter type 4).^121^

In cardiovascular disease, exosome-mediated communication between endothelial and immune cells is emerging as a key player at different stages of disease development.^122^ Cardiac exosomes of endothelial and cardiomyocyte origin locally produced after myocardial infarction were shown to be taken up by monocytes infiltrating the ischemic heart and to subsequently increase their proinflammatory response.^32^ In this context, endothelium-derived EVs were enriched in VCAM-1 (vascular cell adhesion protein 1), localized to the spleen, and interacted with and mobilize splenic monocytes in myocardial infarction.^123^

#### Other Cardiac Exosomes

Recently in their study, Qiao et al compared the treatment effects of cardiac exosomes from healthy donor hearts to those of cardiac exosomes from failing hearts. They demonstrated that pathological condition altered the miRNA composition of cardiac-derived exosomes and impaired their regenerative activities. Importantly, they identified miR-21-5p as a key factor contributing to exosome-mediated heart repair, enhancing angiogenesis and cardiomyocyte survival through the phosphatase and tensin homolog/Akt pathway.^124^

Interestingly, recent studies have identified exosomes as mediators of multiple forms of cardiovascular calcification, in both hyperphosphatemic and inflammatory milieus. Exosomes released by smooth muscle cells, valvular interstitial cells, endothelial cells, and macrophages may promote or inhibit mineralization, depending on the phenotype of their parental cells and extracellular environment to which they are secreted.^125^ Kapustin et al reported that coagulation protein prothrombin can bind to the surface of vascular smooth muscle cell–derived exosomes and can also be loaded into exosomes by cellular internalization therefore regulating coagulation and calcification.^126^

Another potentially important source of exosomes in the heart are immune cells such as dendritic cells or macrophages which are able to mediate immune responses on vesicle secretion. For example, Liu et al reported that exosomes derived from dendritic cells that have infiltrated infarcted myocardium could act as activators of CD4^+^ T cells and improve wound healing postmyocardial infarction.^127^ It has been also reported recently that activated macrophages secrete mir-155–enriched exosomes inhibiting target cardiac fibroblast proliferation and enhancing inflammation during cardiac injury.^128^

Communication between pericytes and other cardiac cell types forms another major crosstalk in myocardial remodeling. When stimulated with hypoxic conditions, pericyte-derived conditioned media modulated proliferation of cardiac fibroblasts and macrophage.^129,130^ In a similar study, activation of the hypoxia-inducible factor pathway in pericytes was essential for the promotion of endothelial cell angiogenesis in an exosome-dependent manner.^129^ Conversely, inflammation-induced endothelial cell–derived EVs regulated expression of angiogenic genes in pericytes.^131^ Taken together, these results suggest that pericytes and endothelial cell communication essentially contribute to remodeling events and the flow of information via exosomes between pericytes and endothelial cells is bidirectional.

#### Immortalized Cell Lines to Scale Up EV Production

Immortalization of cell lines to boost engineered EV production is a topic of great clinical interest as large amounts of EV are needed for clinical applications, eg, after myocardial infarction. Indeed, recently it was shown that genetic manipulation of β-catenin can converted deleterious fibroblasts into therapeutically-beneficial engineered novel cell entities that can produce EVs in high amount and strong functional efficacy.^132^

### Cardiac Exosomes in Long Distance Relationships: Crosstalk Between the Heart and Peripheral Organs

#### Circulating Exosomes

Communication mediated by cardiac exosomes has also been described at distant microenvironments. In this line, recent investigations by Pironti et al performed in mice subjected to pressure overload revealed myocardial secretion of AT1Rs (angiotensin II receptor type 1) via exosomes into the circulation.^133^ These data indicated that functional AT1Rs are carried by EVs elsewhere in circulation, providing a new paradigm to the traditional G protein–coupled receptor trafficking at a whole organism level.^133^ Interestingly, AT1R knockout mice, when administered with AT1R-enriched exosomes, showed improvement in blood pressure responsiveness to angiotensin II, providing functional evidence that exosomes can significantly mediate myocardial signaling and function.^133^ Li et al investigated the role of coronary serum exosomes and showed that exosomes from patients with myocardial ischemia had lower levels of miR-939-5p compared with healthy patients which promoted endothelial angiogenesis through the inducible nitric oxide synthase–nitric oxide pathways.^134^ Importantly, authors demonstrated that cardiomyocytes, but not cardiac fibroblasts or endothelial cells, were the source of bioactive exosomes in coronary serum.^134^ Another recent study found that long-term exercise–derived circulating exosomes conveyed cardioprotective signals and identified exosomal miR-342-5p as a novel cardioprotective bioactive factor against myocardial ischemia/reperfusion injury in rats.^135^

#### SOS Signal to Bone Marrow Cells

A growing interest in understanding the signal exchanges between myocardium and bone marrow led to the conclusion that exosomes may play an important role in these regulatory mechanisms. Interestingly, Cheng et al showed that circulating myocardial microRNAs are released after acute myocardial infarction into the circulation in exosomes and mediate functional crosstalk between the ischemic heart and the bone marrow.^136^ Furthermore, exosomes mediate the transfer of muscle-specific miRNAs to bone marrow mononuclear cells, where muscle-specific miRNAs downregulate *CXCR4* (C-X-C motif chemokine receptor 4) expression and mediate progenitor cell mobilization.^136^

### Releasing the Handbrake on Cardiac Exosome Research

There are several major challenges in studying cell type–specific exosomes and their function in recipient cells locally or in distant target tissues, which we highlight within this paragraph.

#### Lack of Universal Techniques and Standard Protocols for Exosome Isolation and Characterization

All currently used methods for exosome isolation and characterization have different advantages and disadvantages, and although these techniques are commonly used in practice, some issues are still unsolved (Tables 1 and 2). Importantly, some methods cannot distinguish membrane vesicles from nonmembranous particles of similar size while others are not sensitive enough to detect the EVs with a diameter of ≤100 nm.

**Table 2.**
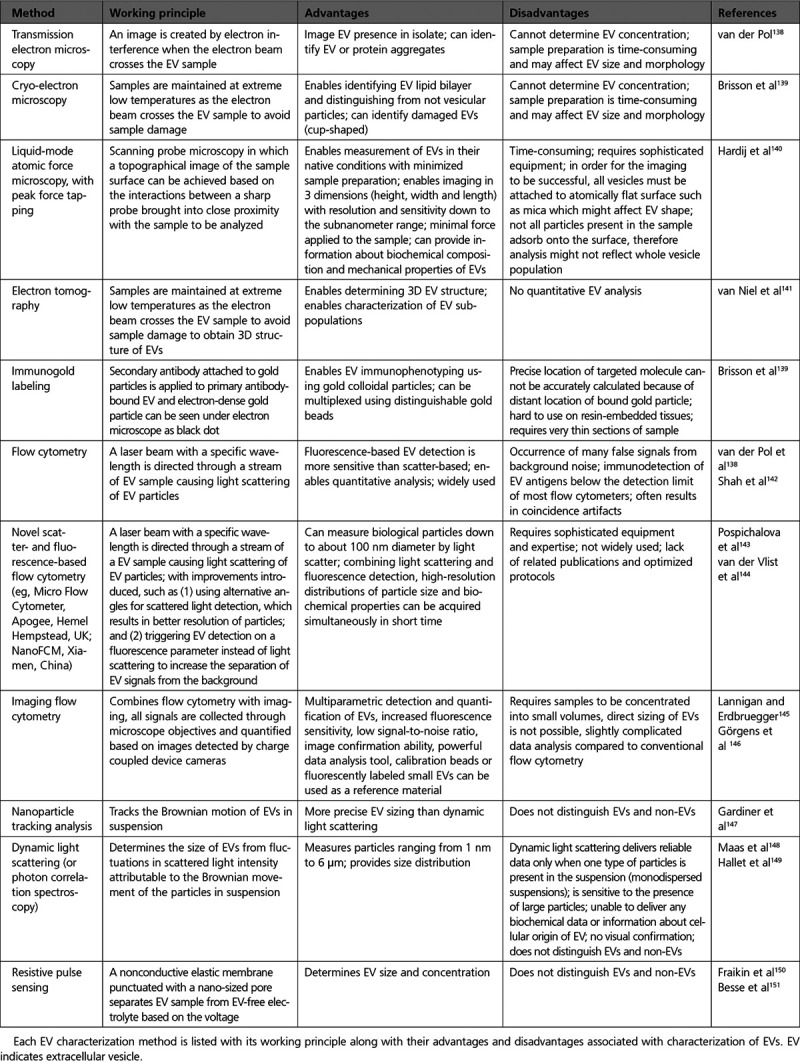
List of EV Characterization Methods

#### Lack of Universal Exosome Markers

Rapid advances in the identification of universal exosome-specific markers as well as parental cell–specific exosome components would enable the enrichment of this particular EV subtype through flow sorting or antibody-based isolation strategies ultimately enhancing our understanding of exosome biology. Importantly, such experimental progress would possibly allow researchers to costain parental cell–specific markers along with EV-specific markers to determine and compare different EV subpopulations secreted by investigated cell type.

#### Unclear Exosome Packaging and Internalization Mechanisms

Mechanisms through which exosomes are loaded with selective molecules are still unclear. Existing evidence on exosome packaging and intracellular trafficking after internalization are mainly obtained from in vitro studies of specific cell types in the culture, which do not truly reflect physiological function. Studies using in vivo models of exosome trafficking are more reliable and will likely identify molecules involved in exosome biogenesis and uptake by recipient cells.

#### Methodological Issues on Exosome Experiments In Vitro and In Vivo

Primary cultures of cardiomyocytes and cardiac fibroblasts, or other cardiac cells do not represent pure cultures, and typically have other unwanted cell types as contaminants. Moreover, commercially available cardiac cell lines as well as iPSC-derived cardiac cells^137^ do not represent the true physiology of cells in the myocardium and thus, often lead to poor interpretation of exosome biological functions. Furthermore, investigations of exosomes in vitro often involve inhibitors of exosome secretion enabling the study specific mechanisms regulating exosome release and clearance. One of the major limitations of these inhibitors is that they are known to affect the entire cellular secretome and other unrelated exosome and nonexosome pathways. Small molecule inhibitors or inducers of exosome-specific proteins that can be effectively used in vivo can shed better understanding of exosome biology in vivo. A major limitation in studying exosome distribution in vivo is the lack of suitable methodologies to investigate cell-specific exosome types secreted within the local tissue environment or into the circulation.

## Shedding a new light on cardiovascular diagnostics and therapeutics

### A Closer Look: Exosome Diagnostics

**Figure 3.**
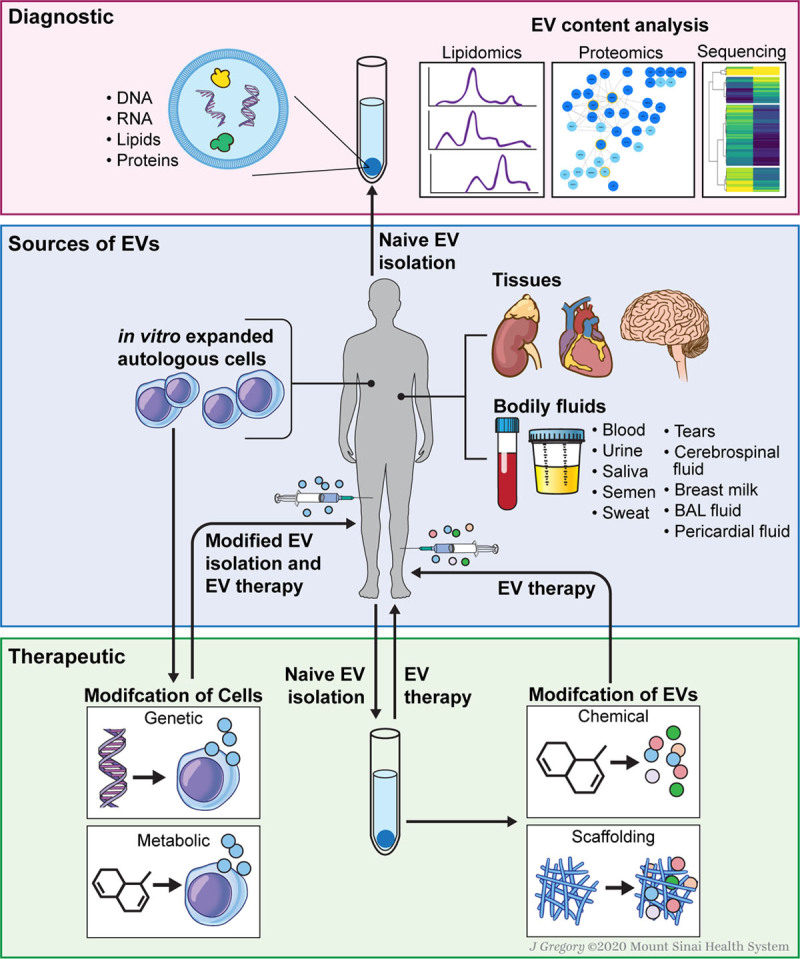
**EV diagnostics and various strategies for EV-based therapies.** Diagnostics: EVs carry bioactive molecules from their host cells that are indicative of pathophysiological conditions, and they are considered to be crucial for biomarker discovery for clinical diagnostics. Therapeutics: EVs can be isolated directly from body fluid and tissue or indirectly from in vitro cultured cells. Cell modification by genetic or metabolic engineering can lead to the release of modified EVs. Alternatively, EVs can be modified directly using chemical treatment or scaffolding strategies. BAL indicates bronchoalveolar lavage; and EV, extracellular vesicles.

Exosomes represent a precious biomedical tool as their molecular content is a fingerprint of the releasing cell type and its physiological state, and because they are detected in easily accessible body fluids such as blood and urine (Figure 3).

#### Circulating Exosomes

Recently, several studies have shown that circulating EVs isolated from patients with cardiovascular disease differ not only in their molecular content compared with healthy subjects, but also in EV counts.^152^ For instance, the amount of circulating microvesicles was found to be elevated in patients with cardiovascular risk factors such as diabetes.^153^ In the context of exosomal content reflecting different pathophysiological stages, recent studies showed that miR-1 and miR-133a serum levels are increased in patients with acute myocardial infarction, Takotsubo cardiomyopathy, and unstable angina pectoris.^154^ In vitro experiments confirmed exosomal origin of these miRNAs as stimulation of H9C2 myoblasts with a calcium ionophore led to elevated miR-133a expression within exosomes with the highest rate when cells were dead. This observation led to the conclusion that miR-133a secretion is connected to cardiac injury and cardiomyocyte death. Furthermore, a cardioprotective miR-214 originating from human endothelial cell–derived exosomes was identified in plasma of coronary artery disease patients and was shown to be upregulated in a mouse model of ischemic injury regulating cardiac survival.^117,155,156^ Interestingly, other studies showed that exosomal miRNAs can be used as biomarkers in early stages of acute myocardial infarction as well as in the prognosis of late stages in heart failure development. Matsumoto et al indeed identified miR-192, miR-194, and miR34a as predictors for heart failure development and ventricular remodeling.^157^

These findings exemplify future prospects for exosomes to serve as diagnostic and prognostic markers. Furthermore, as elevated exosome levels have been shown to correlate with the severity of cardiovascular disorders, a potential approach would be to block exosome formation or secretion, thereby preventing signaling pathways mediated by exosomes.

### A Closer Look: Exosome Therapeutics

#### How to Choose When You Have Too Many Options: Sources of Therapeutic Exosomes

In recent years, attention has been focused on the potential of stem cells to regenerate and repair the injured heart. A large number of studies, including one of the first investigations from our laboratory, are building a consensus that exosomes constitute a key paracrine component that mediate the reparative function of stem cells in the heart.^77,158–161^

Cells extensively studied for exosome-based cardiac repair include mesenchymal stem cells (MSCs),^109,162–165^ cardiac stem cells,^166–168^ and endothelial progenitor cells.^77,158^ Besides multipotent/unipotent adult stem cell–derived exosomes, vesicles released by pluripotent stem cells (ie, embryonic stem cells^160^ and iPSCs^85^) have been similarly investigated in the field of cardiac regenerative medicine. Exosomes mediate their cardioprotective effect often by acting directly on cardiac endothelial cells and promoting angiogenesis.^164,169,170^ Interestingly, vesicles purified from other biological sources, such as body fluids (eg, plasma, pericardial fluid) and somatic cells (eg, cardiomyocytes) were also shown to represent a promising therapeutic option for various cardiovascular diseases.^114,171–174^

Latest research and debates on cardiac repair and regenerative potential of exosomes from a variety of biological sources were discussed in the recent review.^175^ Additionally, in 2018, the European Society of Cardiology Working Group published a position paper providing a set of recommendations for the analysis and translational application of exosomes with the focus on the diagnosis and therapy of the ischemic heart.^176^

#### Messing With Mother Nature: Strategies for Exosome-Based Therapies

To use exosomes as therapeutics, vesicles can either be exogenously reengineered and modified to serve as a drug delivery tool, or can be used as the active drug themselves.

##### Normal Exosome Production

Cells secrete exosomes under normal physiological conditions or in response to an external stimuli.^177^ These vesicles can either be isolated directly from body fluids and tissue explants or primary cell types prepared from them can be indirectly used to collect exosomes from conditioned media after cell culture (Figure 3).

##### Cell or Direct Exosome Modification

Cell manipulations aimed at enhancing beneficial properties of exosomes may include introduction of microenvironmental stimuli such as cell culture at lower oxygen levels (Figure 3). Hypoxia treatment of mouse cardiac progenitor cells resulted in quantitative increase of exosomes secretion with altered exosomal molecular content by selective loading with proangiogenic and anti-fibrotic miRs.^178^ Indeed, hypoxia-induced exosomes modification enhanced exosome-mediated tube formation in endothelial cells, reduced profibrotic gene expression in fibroblasts stimulated with TGF-β (transforming growth factor β) and alleviated cardiac fibrosis with improved cardiac function in a rat model of ischemia/reperfusion injury.^178^ Similarly, exosomes isolated from bone marrow–derived MSCs cultured under hypoxia were enriched with miR-125-5p and hypoxia exosomes treatment facilitated cardiac repair in a mouse model of myocardial infarction.^179^

Using genetic or metabolic engineering of cells or through cellular uptake of exogenous material, exosomes can be modified for function (Figure 3). Genetic engineering can be used to introduce mRNA and noncoding oligonucleotides (eg, miRNA or small interfering RNA) into cells, where nucleic acids can be subsequently packaged into exosomes.^180^ Metabolic labeling involves supplementing cell culture medium with nonnative metabolites to introduce functional groups, such as azides, alkynes, thiols, methacryloyls, and ketones, to exosomes, which allows subsequent bio-orthogonal reactions to be performed.^180^

Exosomes themselves can be chemically or biologically modified to broaden or enhance their therapeutic properties (Figure 3).^180,181^ As an example, vesicle membrane can be permeabilized to allow active loading of molecules into the exosomes. Analogous approach uses lipophilic or amphiphilic molecules that can insert into the exosome membrane via hydrophobic interactions with the phospholipid bilayer.^180^ Exosome surface can also be modified chemically (eg, carbodiimides can be utilized to modify native amines to present azide groups for click chemistry reactions).^180^ In addition, exogenous material can be introduced to exosomes via liposomes or micelles that fuse with cytoplasmic membranes.

##### Exosomes in Tissue Engineering

Combining exosomes with biomaterial scaffolds provides a promising strategy for efficient delivery of exosomes to a specific tissue type as well as to retain exosomes durably at the site of injury (Figure 3). Zhang et al combined exosomes obtained from human iPSC-derived MSCs (hiPSC-MSC-Exos) with tricalcium phosphate, a clinically approved osteoconductive biomaterial.^182^ Using that approach, they repaired critical-sized calvarial bone defects and found that exosome/tricalcium phosphate incorporated scaffolds possessed better osteogenesis activity than pure biomaterial.^182^ In a similar fashion, exosomes obtained from human iPSC-derived MSCs integrated with acellular tissue patch for articular cartilage regeneration retained EVs better and positively regulated both chondrocytes and human bone marrow–derived MSCs in vitro.

While exosome-scaffolds may offer increased therapeutic retention at sites of tissue damage, it may also offer reduced off-target effects, which will need further investigation. Nevertheless, such scaffold-based exosome targeting approaches could provide new means of enhanced therapeutic efficacy for application of exosomes in cardiology.

#### Critical Aspects of Future Cardiac Therapeutics with Exosomes

##### Pros

It is worth emphasizing that nonliving yet bioactive exosomes can exert unique biological activity reflective of their cellular origin that may be potentially applied for cardiovascular therapeutics. Exosomes are smaller, less complex, and more stable than parental cells, and are relatively easy to modify, manufacture, and store.^183^ Owing to their nature, exosomes with a robust membrane may provide a protection against enzymatic and nonenzymatic degradation of their molecular content,^183^ and could have lower tumorigenicity and immunogenicity compared with direct transplantation of cells.^85^

##### Cons

Exosomes are internalized by cells relatively quickly in and around sites of delivery, often entering nonspecific cell types as well as systemic circulation.^160^ Therefore, major challenge in developing exosome-based therapy is to determine whether tailored vesicles targeting specific cell types within the tissue (eg, cardiomyocytes) can be obtained and whether the short half-life of vesicles can be extended.^160^ It is crucial to assess the safety of exosome-based therapy in clinical settings, investigating their biodistribution, toxicity, thrombogenicity, and half-life times in vivo.^111,184^ In this line, the use of large animal models (over rodent models) should be encouraged for preclinical testing of the therapeutic efficacy of exosomes with careful considerations for clinical translation. Furthermore, careful exosome dose and dosage regimen must be determined along with the development of optimal procedures for efficient vesicle administration. Before bringing exosomes to clinic, it is also important to ensure that large-scale supply of exosomes is feasible in recommended safety level meeting the clinical requirement. It will be important to identify a robust source to obtain massive exosome production to increase the overall exosome yield with clinical-grade purity (eg, by genetic modification of producer cells or exosome production in response to external stress).

#### Exosomes in Clinical Trials

Exosomes should go through proper clinical trials to be proven safe and effective before being marketed to patients. A search on https://www.clinicaltrials.gov found 95 interventional clinical trials (both recruiting and not yet recruiting) involving exosomes or extracellular vesicles or microvesicles. The bulk of these exosome clinical trials are occurring within the United States and Europe. Interestingly, few of these studies involve cardiovascular interventions (eg, Role of Exosomes Derived from Epicardial Fat in Atrial Fibrillation [NCT03478410; Sheba Medical Center, Israel]; Allogenic Mesenchymal Stem Cell Derived Exosome in Patients with Acute Ischemic Stroke [NCT03384433; Isfahan University of Medical Sciences, Iran]; and Antiplatelet Therapy Effect on Extracellular Vesicles in Acute Myocardial Infarction [NCT02931045; Medical University of Warsaw, Poland; University of Amsterdam, Netherlands]).

As exosomes become recognized for their benefits as cell-free therapeutics, exosome clinical trials are increasing in number. An example of a published and completed clinical phase II trial can be found in the work done by Besse et al as a continuation of 2 previous phase I trials.^151^ Even though the primary end point was not reached, this clinical trial confirmed the efficacy of injected dendritic cell–derived exosomes in enhancing natural killer cell immune responses in patients with advanced nonsmall cell lung cancer.^151^ Unfortunately, vast majority of exosome clinical trials are still in progress and respective study outcomes are not yet available online or published. Therefore, whether clinical trials involving administration of exosomes will become successful remain to be seen in the near future.

## The future looks bright

Exosomes show great potential to improve both diagnosis and therapy within cardiovascular medicine, but many questions remain unanswered.

### Technical Problems

Because the outcome of any exosome experiment can be biased by choices made (eg, in sample collection, exosome isolation, or storage), it is critical to comprehensively study protocols for exosome isolation. Furthermore, standardizing the morphological, biochemical, and biological characterization methods for exosome preparations, preferably by using a combination of several methods (Table 2), can improve research outcomes toward a better clinical application.

### Get to the Root of It

While exosome-based biomarkers offer great potential in the development of unique signatures for various cardiovascular diseases, a greater understanding of exosome biology is needed before we can draw any strong conclusions. Our knowledge of exosome synthesis, cargo-loading, and uptake pathways are limited, and as our knowledge of the basic science increases, our ability to make use of exosomes in diagnostics and therapeutics should be improved.

### Clinical Applications Are on the Horizon

Studies conducted so far have indicated that exosomes derived from various types of progenitor cells exert cardioprotective and regenerative effects in different experimental settings, yet translation to clinical practice would require further exploration of this phenomenon. Chemical or biological modification strategies offer exciting opportunity to extend therapeutic capability of exosomes beyond their native function, most likely by improving their half-life and efficiency of cargo delivery to target tissues, and maintaining progress particularly in this area may substantially advance the field.

### Light at the End of the Tunnel

Exosome-based therapeutics hold great promise for the development of therapy aimed at regenerating and repairing heart after pathological damage. However, recent excitement and enthusiasm over these powerful cell-free extracellular components should not mask the significant research efforts that still need to be taken to advance our understanding of cardiovascular exosomes and to translate them into the clinic. Despite these challenges, the emerging field of exosome signaling offers many opportunities for cardiovascular medicine.

## Acknowledgments

The authors thank Jill Gregory, Associate Director of Instructional Technology at Icahn School of Medicine, for the help with illustrations (Figures 2 and 3).

## Sources of Funding

This work was supported by National Institutes of Health grants R01HL140469, R01HL124187, and R01HL148786 and New York State Stem Cell Science C32562GG (to S.S.), and by the German Research Foundation (DFG Th903-20-1) and the European Research Council Consolidator grant Longheart (to T.T.).

## Disclosures

Dr Thum has filed and licensed patents in the field of noncoding RNAs; is founder and shareholder of Cardior Pharmaceuticals GmbH; and reports speaker fees or other support from Boehringer Ingelheim, Novo Nordisk, Amicus Therapeutics, Sanofi-Genzyme, and Takeda (outside the scope of this review). Dr Sahoo has filed patents in the field of exosomes for cell and gene therapy. The other authors report no conflicts.
